# Detection of HPV and Human Chromosome Sites by Dual-Color Fluorescence *In Situ* Hybridization Reveals Recurrent HPV Integration Sites and Heterogeneity in Cervical Cancer

**DOI:** 10.3389/fonc.2021.734758

**Published:** 2021-10-05

**Authors:** Jinfeng Xiong, Jing Cheng, Hui Shen, Ci Ren, Liming Wang, Chun Gao, Tong Zhu, Xiaomin Li, Wencheng Ding, Da Zhu, Hui Wang

**Affiliations:** ^1^ Department of Obstetrics and Gynecology, Tongji Hospital, Tongji Medical College, Huazhong University of Science and Technology, Wuhan, China; ^2^ Department of Obstetrics and Gynecology, Zhongnan Hospital, Wuhan University, Wuhan, China

**Keywords:** human papillomavirus, HPV integration, FISH, cervical cancer, site-specific

## Abstract

Human papillomavirus (HPV) integration in the human genome is suggested to be an important cause of cervical cancer. With the development of sequencing technologies, an increasing number of integration “hotspots” have been identified. However, this HPV integration information was derived from analysis of whole cervical cancer tissue, and we know very little about the integration in different cancer cell subgroups or individual cancer cells. This study optimized the preparation of probes and provided a dual-color fluorescence *in situ* hybridization (FISH) method to detect HPV integration sites in paraffin-embedded cervical cancer samples. We used both HPV probes and site-specific probes: 3p14 (*FHIT*), 8q24 (*MYC*), 13q22 (*KLF5*/*KLF12*), 3q28 (*TP63*), and 5p15 (*TERT*). We detected HPV signals in 75 of the 96 cases of cervical cancer; 62 cases showed punctate signals, and 13 cases showed diffuse punctate signals. We identified 3p14 as a high-frequency HPV integration site in 4 cervical cancer cases. HPV integration at 8p14 occurred in 2 cases of cervical cancer. In the same cervical cancer tissue of sample No.1321, two distinct subgroups of cells were observed based on the HPV probe but showed no difference in cell and nucleus morphology. Our study provides a new method to investigate the frequent HPV integration sites in cervical cancer and reports the heterogeneity within cervical cancer from the perspective of HPV integration.

## Introduction

Cervical cancer remains the leading cause of gynecological tumor-related mortality worldwide and the second most common malignancy in women, with 570,000 women diagnosed with cervical cancer and 311,000 dying from the disease each year (1, 2).

Most HPV infections are cleared by the immune system, but in some cases, the infection persists. Persistent infection with high-risk HPV leads to cervical intraepithelial neoplasia (CIN), which occasionally develops into cervical cancer (3). High-risk HPV types include 16, 18, 31, 33, 58 and other subtypes. HPV16 and 18 infection is common and can be detected in approximately 70% of cervical cancer cases. After infection, the virus can remain in the episome or integrate into the human genome, and the two patterns may coexist (4).

Recent studies have suggested that the integration of HPV in the human genome is an important cause of cervical cancer. Integration can preserve the upstream regulatory region (URR) of the virus. This HPV replication initiation region in the human genome is not stable but activates the cell’s DNA replication and other systems, which is an important “trigger” for cancer (5). The integration of HPV DNA into the human genome causes various genetic changes, such as oncogene amplification, inactivation of tumor suppressor genes, chromosomal rearrangement, and genomic instability. Integration can occur near key genes, leading to increased instability near integration sites in the human cell genome, resulting in local chromatin structural and functional changes and even the formation of new virus-human gene fusion transcripts in response to URRs of the virus (6, 7). In 2015, whole-genome sequencing and high-throughput virus capture sequencing methods identified up to 3,667 HPV integration breakpoints in cervical neoplasms (8). Frequent integration has been reported in genes associated with tumor progression, such as the oncogene MYC. It has been reported that loss of function of the tumor suppressor gene RAD51B after HPV DNA insertion affects DNA repair pathways and genomic instability in tumors (9).

Increasing research on HPV integration has led to the recognition of HPV integration status as a potential biomarker for the prediction of diagnosis, progression, and survival and even as a biomarker for cancer screening (10). With the development of high-throughput sequencing technologies (e.g., whole-genome sequencing, transcriptome sequencing, HIVID, etc.), an increasing number of integration “hotspots” have been identified (8, 9). However, this HPV integration information was derived from analyses of the whole cervical cancer tissue, and we know very little about the integration in different cancer cell subgroups or individual cancer cells. To visually display the more refined HPV integration at the single tumor cell level, this study optimized the preparation of probes and provided a dual-color FISH method to verify the HPV integration sites in paraffin-embedded cervical cancer samples. By preserving the original morphology and spatial structure of tumor tissue, we provide a new method to investigate the relationship between HPV integration and cervical carcinogenesis.

## Materials And Methods

### Tissue Material

Formalin-fixed and paraffin wax-embedded cervical cancer samples were selected from the Department of Pathology, Tongji Hospital, Wuhan, China. All the samples were reviewed and confirmed independently by two pathologists, and cases with discrepancies were discussed until a consensus was reached. Samples with poor morphology or too many lymphocytes were excluded.

Clinical characteristics were obtained from patient charts. The TNM staging system, which is based on surgical and pathological reports, was used in this study to evaluate cervical cancer patients. We collected a total of 96 samples: 93 squamous cell carcinoma (SCC) samples, 3 adenocarcinoma (AD) samples. The study was approved by the hospital’s ethics committee.

### Probe Selection and Labeling Procedures

Bacterial artificial chromosome (BAC) plasmids for 8q24 (RP11-1145O20), 3p14 (RP11-191B8), 3q28 (RP11-373I6), 5p15 (RP11-326E20) and 13q22 (RP11-179I20) were purchased from Life Technologies (California, America). The whole-genome plasmid of HPV types 16 and 18 was a gift from Haraud zur Hausen. The probes were labeled by standard nick translation with biotin- or digoxigenin-dUTPs. The biotin-labeled HPV probes and digoxigenin-labeled BAC probes were then coprecipitated with human Cot-1 DNA and salmon sperm DNA. Pelleted probes were then dissolved in a hybridization buffer composed of 50% formamide, 2×SSC, and 10% dextran sulfate. The detailed protocol and parameters of HPV-BAC dual-color probes are provided in the **Supplementary Material**.

### Tissue Pretreatment

Four-micrometer thick paraffin wax tissue sections were dewaxed, dehydrated with 100% ethanol for 3 min twice before air drying, and pretreated with 3% H_2_O_2_ for 10 min at room temperature. The slides were incubated in 1 M NaSCN for 20 min at 80°C, followed by digestion with 4 mg/ml pepsin (1:3000, Sigma) in 0.02 M HCl for 12 min at 37°C. The slides were rinsed 2 times in 2× SSC, postfixed in 4% formaldehyde for 10 min at room temperature, and dehydrated in an ascending ethanol series.

Probes and target DNA were denatured simultaneously for 7 min at 90°C before hybridization overnight at 37°C. After hybridization, the preparations were washed stringently in 50% formamide (3 × 5 min) and 2× SSC at 43°C.

### Probe Detection

The biotin- and digoxigenin-labeled probes were detected consecutively using the dual-color tyramide signal amplification (TSA) procedure. The biotin-labeled probe was detected by streptavidin-HRP (1:100, Perkin Elmer). Then, the first amplification reaction was carried out under a coverslip by applying 50 μl of Cy3-tyramide (1:50, Perkin Elmer) for 20 min at room temperature. Thereafter, the slides were soaked in blocking reagent (Perkin Elmer) for 15 min at room temperature to block the remaining peroxidase activity. Subsequently, the digoxigenin-labeled probe was detected by anti-digoxigenin-HRP (1:200, Roche), followed by TSA amplification using FITC-tyramide (1:50, Perkin Elmer). Finally, the slides were washed in 4× SSC, dehydrated in an ascending ethanol series, and mounted in Vectashield (Vector Laboratories).

### Microscopic Imaging and Evaluation of FISH Results

Images were recorded with a fluorescence microscope (Olympus BX53) equipped with FITC, TRITC, and DAPI bandpass filters. A minimum of 100 nuclei in each sample were observed for HPV integration site evaluation. Among the cells containing HPV signals, the ratio of cells with colocalized HPV probe signals and BAC probe signals was calculated. Samples with a ratio greater than 60% were evaluated for HPV integration in a specific site.

### Control

HPV and BAC probe hybridization on HPV-positive cell lines (SiHa, HeLa and CaSki) was used as a control for HPV integration site detection. On sample tissue sections, BAC probes were also used as a control for effective hybridization.

## Results

### HPV Integration in Cervical Cancer Cell Lines and Cervical Cancer Tissues Can Be Detected by Dual-Color Fluorescence *In Situ* Hybridization

**Figure 1:**
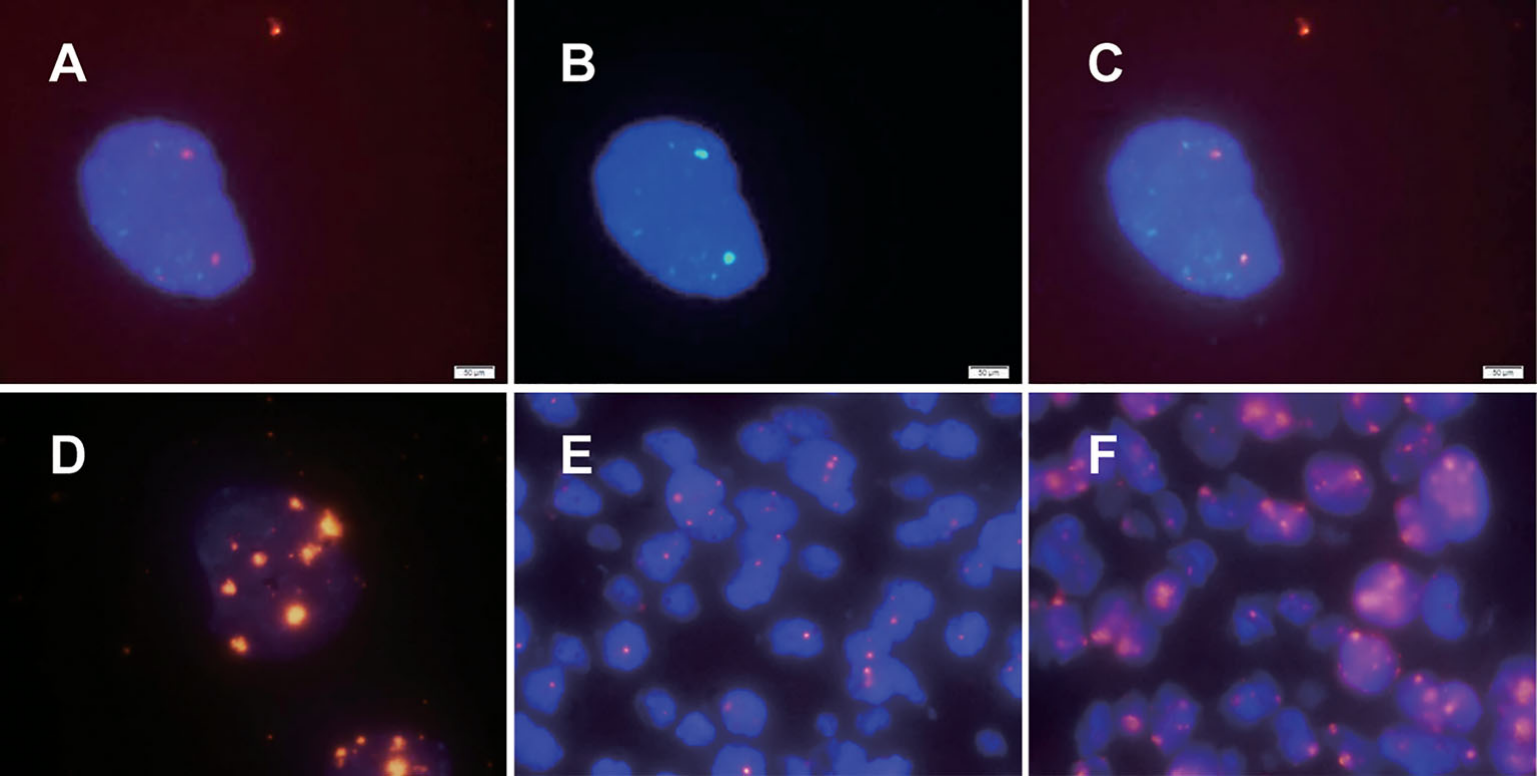
HPV signal patterns in cervical cancer cell lines and cervical cancer paraffin sections detected by dual-color FISH. The fluorescence intensity and area of the HPV signal were observed to increase with increasing HPV integrated copy number in cells, but the relationship was not linear. HPV signals in SiHa and CaSki cells are illustrated in **(A, D)**, respectively. **(B)** The specific chromosomal site (Bacterial artificial chromosome, BAC) probe targeting the 13q22 in SiHa. As shown in **(C)**, the HPV signal and the specific chromosomal site probe targeting the 13q22 (RP11-315L12) signal were colocalized. There were two main types of HPV signals in paraffin specimens of cervical cancer: punctate signals and diffuse-punctate signals. The punctate signals indicate integrated HPV **(E)**, while diffuse signals indicate the presence of episomal HPV at the same time **(F)**.

HPV16/18 probes were hybridized in SiHa and CaSki cells, two smaller HPV signals were observed in SiHa cells with only two copies of HPV16 (**Figure 1A**), and 7 to 8 signals of different sizes were observed in dots and clumps in CaSki cells with 500 HPV16/18 copies (**Figure 1D**). We observed that with the increase in HPV integrated copy number in cells, the fluorescence intensity and area of the HPV signal also increased, but the relationship was not linear. The results of hybridization in SiHa cells prove that our HPV probe has sufficient sensitivity.

In previous studies, only HPV probes were used to study the presence and amount of HPV integration. To further study the integration sites of HPV, we explored the simultaneous hybridization of HPV probes and BAC plasmid probes into cervical cancer cells. Since previous studies suggested that two HPV16 copies of SiHa were integrated near the KLF5 gene at 13q22, our experiment showed that the HPV signal and the BAC plasmid probe targeting the 13q22 (RP11-315L12) signal were colocalized, as expected (**Figures 1A–C**). Chromosomal site probes can be used to indicate whether HPV is integrated at the site and can be used as a control for whether hybridization is successful. The results from SiHa cells confirmed the credibility of our dual-color probe.

We further explored the application of dual-color probes in paraffin sections of cervical cancer to prepare for possible clinical application. The *in situ* hybridization methods (including experimental procedures and various parameters) in cultured cells and paraffin sections are completely different. Through a review of the literature and experiments (11–13), we confirmed that both the sodium bisulfite and sodium thiocyanide methods are feasible. We found that the sodium bisulfite method was milder and preserved the nucleus well, but it often failed in aged paraffin sections. The sodium thiocyanide method is not easy to use, but the treatment is more complete, and it also has a higher success rate of hybridization for aged paraffin sections. To study the integration sites of HPV, we used the sodium thiocyanide method in this study. There were two main types of HPV signals in cervical cancer paraffin sections: punctate signals and diffuse-punctate signals. The punctate signal suggests integrated HPV (**Figure 1E**), and the diffuse signal suggests the presence of episomal HPV at the same time (**Figure 1F**).

### Integration of HPV at Human Chromosomal Loci in Cervical Cancer Tissues

We detected HPV signals in 75 cases of cervical cancer; 62 cases showed punctate signals, and 13 cases showed diffuse punctate signals (**Table 1**). We did not observe a simple diffuse signal in cervical cancer, which is consistent with the findings that HPV is generally integrated in cervical cancer in previous studies (12, 14).

**Table 1:** Detailed information on HPV integration sites detected by FISH.

Sample ID	Pathology	Stage	HPV16/18		HPV integration	Chromosomal site				
			Punctate	Diffuse		8q24	3p14	3q28	5p15	3q22
1005	SCC	II	▲			2%			2%	
1007	SCC	II	▲			9%		17%	5%	
1009	SCC	I	▲			19%	17%	47%	11%	10%
1011	SCC	II	▲			7%		6%	2%	10%
1013	SCC	I	▲		3p14	3%	**88%**	7%	24%	4%
1015	SCC	I	▲		5p15			6%	**99%**	9%
1017	SCC	II	▲			2%				5%
1023	SCC	I	▲			9%				
1025	SCC	II	▲			4%	3%	28%	26%	8%
1053	SCC	I	▲				3%	13%	6%	5%
1055	SCC	I	▲				2%		11%	7%
1071	SCC	I	▲					5%		2%
1073	SCC	I	▲	▲				7%	3%	13%
1075	SCC	II	▲	▲		10%	11%	11%	2%	5%
1083	SCC	I	▲	▲		19%	22%	6%	15%	6%
1087	SCC	II	▲					10%		6%
1095	SCC	I	▲							4%
1097	SCC	I	▲					3%	7%	4%
1101	SCC	II	▲			4%			5%	
1103	SCC	I	▲	▲		10%	11%	8%	24%	
1111	SCC	I	▲	▲		11%	6%	10%	8%	3%
1117	SCC	II	▲				6%			
1123	SCC	I	▲					14%		4%
1135	AD	I	▲							
1141	SCC	I	▲						8%	3%
1142	SCC	I	▲			8%		27%	6%	
1143	SCC	II	▲			7%				
1151	SCC	II	▲		3p14		**67%**	16%	16%	
1162	SCC	I	▲			3%	4%			2%
1166	SCC	II	▲					4%	13%	11%
1168	SCC	II	▲					13%		
1169	SCC	I	▲	▲				12%	9%	
1173	SCC	II	▲		13q22			10%	13%	**83%**
1174	SCC	I	▲					7%	14%	8%
1175	SCC	II	▲						8%	
1293	SCC	III	▲		8q24	**100%**	8%		14%	18%
1295	SCC	I	▲	▲		6%	6%	21%	8%	18%
1297	SCC	I	▲				13%		12%	
1299	SCC	I	▲	▲		2%	5%	6%		9%
1301	SCC	I	▲				2%	4%	14%	10%
1303	SCC	III	▲	▲		2%	11%	12%	5%	26%
1305	SCC	II	▲			15%	3%		13%	
1307	SCC	I	▲							5%
1309	SCC	I	▲	▲				3%	14%	7%
1311	SCC	I	▲			9%			6%	23%
1313	AD	II	▲			10%		35%		12%
1317	SCC	II	▲				7%	5%	7%	
1319	SCC	I	▲					4%	10%	6%
1321	SCC	I	▲			5%		20%	27%	5%
1325	SCC	II	▲		3p14		**71%**	14%	11%	9%
1329	SCC	I	▲			6%	26%	1%	3%	5%
1331	SCC	II	▲				3%	26%	8%	5%
1335	SCC	I	▲				5%	15%	5%	4%
1339	SCC	II	▲				4%	13%	2%	20%
1342	SCC	I	▲		8q24	**67%**	3%		16%	
1343	SCC	I	▲			20%	13%	29%	14%	8%
1344	SCC	I	▲				4%	2%	3%	9%
1345	SCC	I	▲					8%	3%	5%
1346	SCC	III	▲	▲		12%	16%	5%	20%	17%
1347	SCC	I	▲	▲		27%		7%	6%	24%
1348	SCC	I	▲					2%	9%	16%
1351	SCC	I	▲				4%		19%	2%
1352	SCC	I	▲							7%
1353	SCC	II	▲						5%	
1356	SCC	IV	▲					6%	12%	11%
1357	SCC	III	▲				3%	5%	18%	7%
1360	SCC	I	▲				6%	1%	7%	17%
1361	SCC	II	▲			6%	7%	2%	2%	10%
1362	SCC	II	▲				3%		17%	
1363	SCC	II	▲			4%	7%	9%	15%	17%
1364	SCC	II	▲				9%		5%	3%
1365	SCC	I	▲						14%	
1366	SCC	II	▲				5%			6%
1367	SCC	I	▲		3p14		**82%**	3%	9%	4%
1368	SCC	I	▲				2%		23%	2%

SCC, squamous cell carcinoma; AD, adenocarcinoma. The bold values indicate ratio more than 60%.

▲ HPV16 positive and/or HPV18 positive.

**Figure 2:**
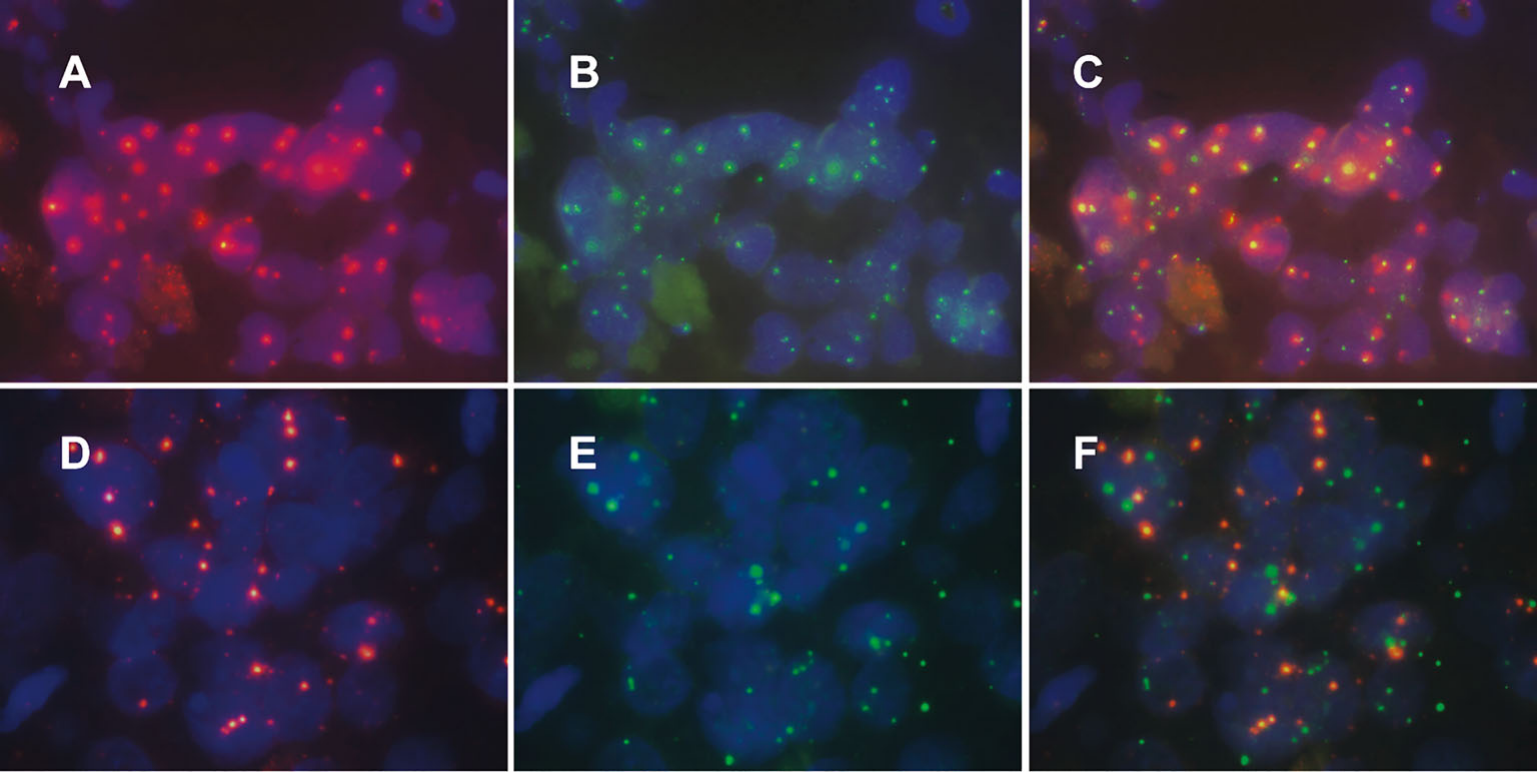
Patterns of human specific chromosomal site probe signals and HPV signals in cervical cancer tissue. Among the cells containing HPV signals, the ratio of cells with colocalized HPV probe signals and specific chromosomal site probe signals was calculated. Samples with a ratio greater than 60% were evaluated for HPV integration in a specific site **(A–C)**; otherwise, we evaluate that HPV was not integrated at this site **(D–F)**.

Our criteria for determining HPV integration were as follows: when the percentage was greater than 60% (cells showing colocalized HPV signals and chromosomal locus probe signals/cells containing HPV signals), we concluded that HPV was integrated at this site (**Figures 2A–C**); otherwise, there was no integration at this site (**Figures 2D–F**). Based on a review of the literature on HPV integration sites and genomic alterations in cervical cancer (8, 15), we prepared probes targeting five regions of the human chromosome: 3p14 containing *FHIT*, 8q24 containing *MYC*, 13q22 containing *KLF5*/*KLF12*, 3q28 containing *TP63*, and 5p15 containing *TERT*. We detected 3p14 as a high-frequency HPV integration site in 4 cervical cancers. HPV integration at 8p14 occurred in 2 cases of cervical cancer. We found HPV integration in one patient each at 5p15 and 13q22. We did not detect HPV integration at 3q28, although 3q28 shows the greatest copy number amplification in cervical cancer and is associated with CIN progression (16) (**Table 1**).

### HPV Signaling Suggests Clonality and Heterogeneity in Cervical Cancer Tissue

**Figure 3:**
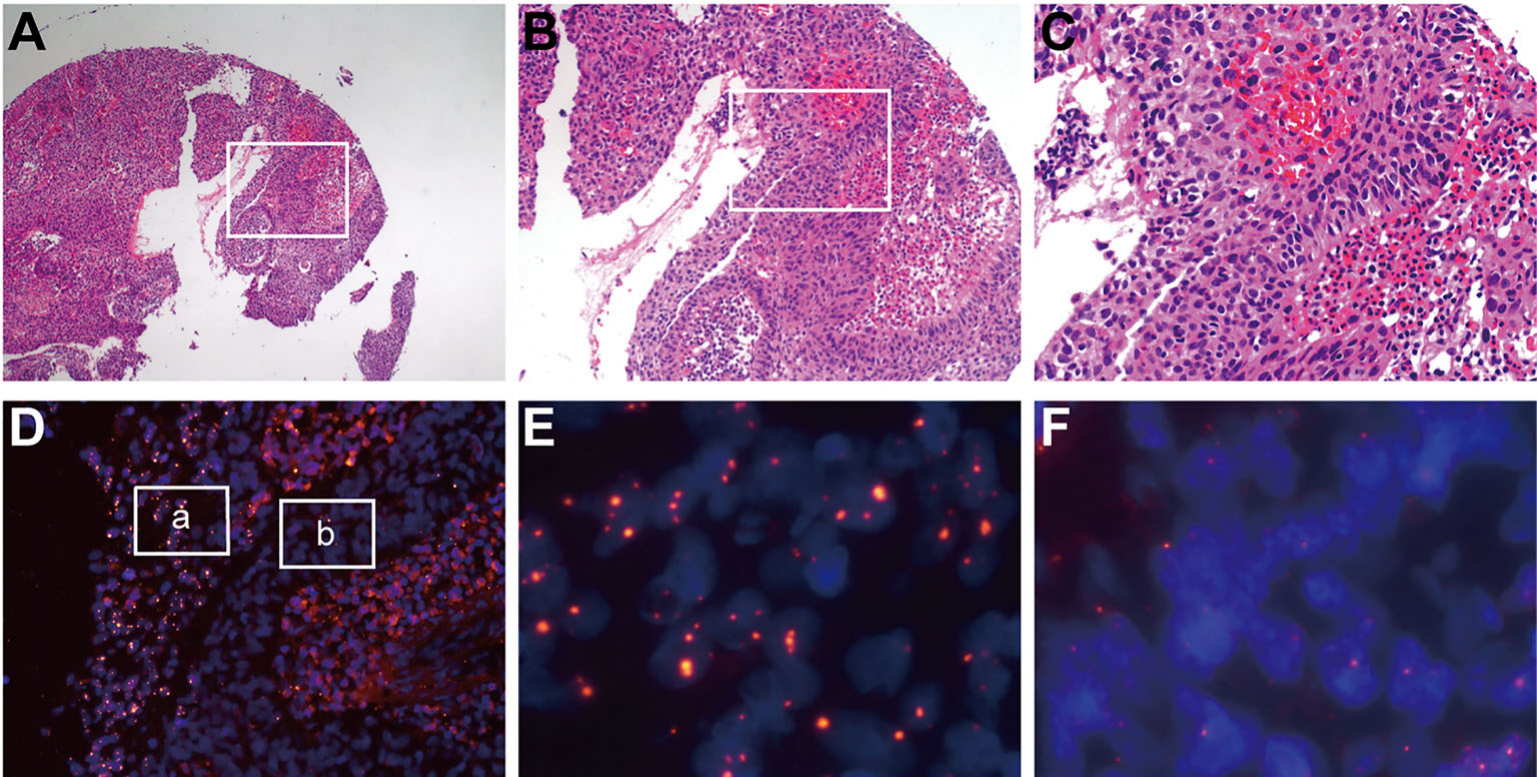
HPV signaling suggests clonality and heterogeneity in cervical cancer tissue. In sample No. 1321, two groups of cancer cells in the same cervical cancer tissue showed no difference in cell and nuclear morphology after HE staining and were pathologically identified as the same type of cells **(A–C)**, which were distinguished significantly by HPV probes **(D)**. Every cell in the “a” group **(E)** showed HPV integration, the HPV signal area was large, and the intensity was high, suggesting intense HPV integration. In the “b” group **(F)**, some of the cells showed HPV integration, the HPV signal area was small, and the intensity was low, suggesting a low integration.

In contrast to PCR or high-throughput sequencing methods, the FISH method can detect HPV integration without destroying tissue morphology. Using an HPV probe as a guide, we observed major differences (both clonality and heterogeneity) in cancer cells within cervical cancer. For example, in sample No. 1321, two groups of cancer cells in the same cervical cancer tissue showed no difference in cell and nucleus morphology after HE staining and were pathologically identified as the same type of cells (**Figures 3A–C**). However, two distinct subgroups of cells were observed by the HPV probe. Every cell in the a group showed HPV integration, the HPV signal area was large, and the intensity was high, suggesting intense HPV integration. In the b group, some of the cells showed HPV integration, the HPV signal area was small, and the intensity was low, suggesting low integration (**Figures 3D–F**).

## Discussion

Currently, many studies have suggested that HPV integration plays an important role in the development of cervical cancer, but our knowledge of HPV integration is still insufficient. The reasons are as follows: First, HPV integration sites in the human genome are numerous and scattered, and different studies have reported different integration hotspots (6, 8, 17–20). Previous studies have detected high-frequency HPV integration sites and attempted to explain the relationship between HPV integration and the progression of cervical cancer by using high-frequency HPV integration sites. There is no conclusion yet regarding whether HPV integration is the driver of cervical cancer or the consequence of human cell genome instability during cervical cancer development (21). Second, the HPV sequence is not completely integrated into the human cell genome; only fragments are integrated (14). The integrated fragments are different in different samples and different at different sites in the same sample (8). Previous studies suggested that integrated HPV breaks in the E2 region and retains the viral oncogenes E6 and E7 (21), but the results of high-throughput sequencing suggest that this is not always the case (8). It is still unclear whether every integrated HPV fragment has a role. To elucidate the relationship between HPV integration and cervical cancer and the carcinogenic mechanism of HPV integration, we must be able to truly and accurately detect HPV integration.

Previous studies on HPV integration mainly include PCR, high-throughput sequencing, and *in situ* hybridization, each of which has advantages and disadvantages. PCR technology includes DIPS-PCR, APOT-PCR and others, which use specially designed primers to amplify HPV-human fusion fragments (22, 23). Considering the different specificities of primers, PCR can indeed detect some HPV-human fusion fragments, but some will be missed. High-throughput sequencing technologies, including WGS, RNA-seq, and HIVID, are more efficient and comprehensive in detecting HPV-human fusion fragments (8, 9). High-throughput sequencing technology provides an overall picture of HPV integration in a given tissue. There is a lack of information on differences in HPV integration in the tissue, and validation by other techniques (PCR, FISH) is required.

Previous studies on HPV integration using FISH techniques mainly investigated the association between HPV integration and CIN progression (12, 24). At present, there are few studies on the simultaneous detection of HPV integration and a specific chromosomal locus in cervical cancer paraffin specimens. The main obstacle is the preparation of FISH probes. The specific chromosomal site probe was nick translated from the BAC clones. The BAC was sufficiently long, and even if HPV integration led to the loss of some chromosomal fragments, the chromosomal site-specific probes still showed signals, such as at 3p14. Therefore, regardless of whether the DNA copy number at the integration site increases or decreases, we can effectively detect the HPV integration site with colocalization of two colors.

The advantage of paraffin specimens over primary cultured cancer cells is that paraffin specimens are easier to obtain and retrospectively analyzed, can preserve the morphology of cervical cancer tissue, and can be applied clinically. To simultaneously detect integrated HPV and a specific chromosomal site, our dual-color FISH technique needs to address the following two key points: probe nick translation and hybridization process. The formula of the dual-color probe, time of nick translation, and dose of human COT-1 DNA were optimized through repeated trials in this study. We provide the detailed protocol in the **Supplementary Material** to help more researchers use this method. *In situ* hybridization of paraffin sections is an improvement over previous research techniques (11, 12).

No HPV16/18 signal was detected in 21 cervical cancer specimens, the possible reasons for not detecting HPV16/18 signals in some samples are as follows: (1) There was no HPV integration in this sample. (2) As 80% of cervical cancers are infected by HPV16/18, we selected HPV16/18 probe. However, a few samples may not be HPV16/18 integrated but integrated by other subtypes. (3) Considering the intra-tumoral heterogeneity of cervical cancer tissues, some tumor cell clones in the same tissue have HPV integration but some clones do not. The tumor cell population at the paraffin sections we examined may happened to be with no HPV integration.

We used a threshold value for identifying an HPV integration site: in cells containing HPV signals, the ratio of cells with colocalized HPV signals and chromosomal site-specific signals is more than 60%, which is interpreted as HPV integration at a specific site. The application of FISH in fusion gene research could be used as a reference, since fusion gene and HPV integration have similarities. Tomlins used FISH to evaluate the TMPRSS2:ETV1 fusion gene in paraffin sections of prostate cancer and found that, fusion signals were observed in an average of 31% of the 100 cancer cells in the positive case (Supporting Online Material page 3) (25). Therefore, the cut-off value of 60% in this study is a relatively strict standard. Considering that we are the first to evaluate HPV integration using the ratio of cells with colocalization signals, the threshold of this ratio is worthy of further study.

Our selection of five candidate chromosomal sites is based on a review of HPV integration in cervical cancer research (8, 15). *FHIT* is a tumor suppressor at 3p14, which is significant copy number loss in cervical cancer, as loss of its activity results in replication stress and DNA damage. *MYC* is an oncogene, and it has been suggested that *MYC* activation is associated with HPV integration at 8q24. *KLF5* is a transcriptional activator at 13q22 and may play a role in cell proliferation. Telomerase expression plays a role in cellular senescence, and deregulation of telomerase expression is found in somatic cells. 3q28 is significant copy number gain in cervical cancer, amplification and overexpression of *TP63* at 3q28 is a biomarker of progression from CIN to cervical cancer. In our study, 3p14 and 8q24 were two high-frequency sites of HPV integration. Future studies may be able to reclassify cervical cancer at the molecular level from the perspective of HPV integration.

Moreover, the integrated HPV signal can be used as a guide for the discovery of cervical cancer cell subgroups with different integrated viral loads. These subgroups may reveal the origin, metastasis, and recurrence of cervical cancer. This finding suggests that previous PCR and high-throughput sequencing methods for detecting HPV integration sites in entire cervical cancer tissue cannot accurately reveal the characteristics of different subgroups of cancer cells. This limitation may be why previous studies often report many integration sites, but they cannot determine whether these integration sites are the initiating factors before carcinogenesis or the consequence of genome instability after carcinogenesis.

In clinical applications, high-throughput sequencing technologies such as WGS and HIVID can be combined with FISH to detect HPV integration after cervical cancer tissues are obtained by surgery. On the one hand, FISH can be used to verify sequencing results. Since the determination of HPV integration sites by sequencing is based on the similarity of base sequences, sequencing reads with low specificity or containing repetitive sequences will lead to sequence alignment errors, which can be verified by FISH. On the other hand, FISH can be used to detect whether the cancer cells containing a specific HPV integration site are the majority or the minority, which can be indirectly shown by the abundance of the human-virus fusion sequence in the sequencing results, while it can be intuitively observed by FISH. If cancer cells containing a specific HPV integration site are the majority in the tissue, HPV integration at this site may be the key factor promoting cervical cancer. If they are the minority, HPV integration at this site may be a consequence of genome instability in cancer cells.

In conclusion, our study provides a method for the detection of HPV integration sites in paraffin-embedded cervical cancer samples using dual-color FISH and reports the heterogeneity within cervical cancer from the perspective of HPV integration. Our study provides new methods and ideas for research on HPV integration in cervical carcinogenesis.

## Data Availability Statement

The original contributions presented in the study are included in the article/**Supplementary Material**. Further inquiries can be directed to the corresponding authors.

## Ethics Statement

The studies involving human participants were reviewed and approved by Ethics Committee of Tongji Hospital. The patients/participants provided their written informed consent to participate in this study.

## Author Contributions

HW and DZ designed and supervised the research together with JX. DZ and HS provided technical support. DZ, JX, CR, LW, and WD performed the experiments. CG, TZ, and XL obtained specimens. JC collected clinical data. HW, DZ, JX, and HS provided analysis and interpretation of data. The manuscript was drafted by DZ and JX. All authors contributed to the article and approved the submitted version.

## Funding

This work was supported by funds from National Natural Science Foundation of China (81830074, 81974412, 81772786, 81502253, 81902667 and 82002763).

## Conflict of Interest

The authors declare that the research was conducted in the absence of any commercial or financial relationships that could be construed as a potential conflict of interest.

## Publisher’s Note

All claims expressed in this article are solely those of the authors and do not necessarily represent those of their affiliated organizations, or those of the publisher, the editors and the reviewers. Any product that may be evaluated in this article, or claim that may be made by its manufacturer, is not guaranteed or endorsed by the publisher.

## References

[1] Ferlay J Soerjomataram I Dikshit R Eser S Mathers C Rebelo M. Cancer Incidence and Mortality Worldwide: Sources, Methods and Major Patterns in GLOBOCAN 2012. Int J Cancer (2015) 136:E359–86. doi: 10.1002/ijc.29210 25220842

[2] Bray F Ferlay J Soerjomataram I Siegel RL Torre LA Jemal A. Global Cancer Statistics 2018: GLOBOCAN Estimates of Incidence and Mortality Worldwide for 36 Cancers in 185 Countries. CA Cancer J Clin (2018) 68:394–424. doi: 10.3322/caac.21492 30207593

[3] Crosbie EJ Einstein MH Franceschi S Kitchener HC. Human Papillomavirus and Cervical Cancer. Lancet (2013) 382:889–99. doi: 10.1016/S0140-6736(13)60022-7 23618600

[4] Oyervides-Munoz MA Perez-Maya AA Rodriguez-Gutierrez HF Gomez-Macias GS Fajardo-Ramirez OR Trevino V. Understanding the HPV Integration and its Progression to Cervical Cancer. Infect Genet Evol (2018) 61:134–44. doi: 10.1016/j.meegid.2018.03.003 29518579

[5] Kadaja M Isok-Paas H Laos T Ustav E Ustav M. Mechanism of Genomic Instability in Cells Infected With the High-Risk Human Papillomaviruses. PloS Pathog (2009) 5:e1000397. doi: 10.1371/journal.ppat.1000397 19390600 PMC2666264

[6] Kraus I Driesch C Vinokurova S Hovig E Schneider A von Knebel Doeberitz M. The Majority of Viral-Cellular Fusion Transcripts in Cervical Carcinomas Cotranscribe Cellular Sequences of Known or Predicted Genes. Cancer Res (2008) 68:2514–22. doi: 10.1158/0008-5472.CAN-07-2776 18381461

[7] Lu X Lin Q Lin M Duan P Ye L Chen J. Multiple-Integrations of HPV16 Genome and Altered Transcription of Viral Oncogenes and Cellular Genes Are Associated With the Development of Cervical Cancer. PloS One (2014) 9:e97588. doi: 10.1371/journal.pone.0097588 24992025 PMC4081011

[8] Hu Z Zhu D Wang W Li W Jia W Zeng X. Genome-Wide Profiling of HPV Integration in Cervical Cancer Identifies Clustered Genomic Hot Spots and a Potential Microhomology-Mediated Integration Mechanism. Nat Genet (2015) 47:158–63. doi: 10.1038/ng.3178 25581428

[9] Ojesina AI Lichtenstein L Freeman SS Pedamallu CS Imaz-Rosshandler I Pugh TJ. Landscape of Genomic Alterations in Cervical Carcinomas. Nature (2014) 506:371–5. doi: 10.1038/nature12881 PMC4161954 24390348

[10] Liu L Ying C Zhao Z Sui L Zhang X Qian C. Identification of Reliable Biomarkers of Human Papillomavirus 16 Methylation in Cervical Lesions Based on Integration Status Using High-Resolution Melting Analysis. Clin Epigenet (2018) 10:10. doi: 10.1186/s13148-018-0445-8 PMC5781301 29410710

[11] Hopman AH Kamps MA Smedts F Speel EJ Herrington CS Ramaekers FC. HPV *In Situ* Hybridization: Impact of Different Protocols on the Detection of Integrated HPV. Int J Cancer (2005) 115:419–28. doi: 10.1002/ijc.20862 15688369

[12] Hopman AHN Smedts F Dignef W Ummelen M Sonke G Mravunac M. Transition of High-Grade Cervical Intraepithelial Neoplasia to Micro-Invasive Carcinoma Is Characterized by Integration of HPV 16/18 and Numerical Chromosome Abnormalities. J Pathol (2004) 202:23–33. doi: 10.1002/path.1490 14694518

[13] Algeciras-Schimnich A Policht F Sitailo S Song M Morrison L Sokolova I. Evaluation of Quantity and Staining Pattern of Human Papillomavirus (HPV)-Infected Epithelial Cells in Thin-Layer Cervical Specimens Using Optimized HPV-CARD Assay. Cancer Cytopathol (2007) 111:330–8. doi: 10.1002/cncr.22946 17724679

[14] Pett M Coleman N. Integration of High-Risk Human Papillomavirus: A Key Event in Cervical Carcinogenesis? J Pathol (2007) 212:356–67. doi: 10.1002/path.2192 17573670

[15] Wentzensen N Vinokurova S von Knebel Doeberitz M. Systematic Review of Genomic Integration Sites of Human Papillomavirus Genomes in Epithelial Dysplasia and Invasive Cancer of the Female Lower Genital Tract. Cancer Res (2004) 64:3878–84. doi: 10.1158/0008-5472.CAN-04-0009 15172997

[16] Zhu D Jiang XH Jiang YH Ding WC Zhang CL Shen H. Amplification and Overexpression of TP63 and MYC as Biomarkers for Transition of Cervical Intraepithelial Neoplasia to Cervical Cancer. Int J Gynecol Cancer (2014) 24:643–8. doi: 10.1097/IGC.0000000000000122 24662128

[17] Melsheimer P Vinokurova S Wentzensen N Bastert G von Knebel Doeberitz M. DNA Aneuploidy and Integration of Human Papillomavirus Type 16 E6/E7 Oncogenes in Intraepithelial Neoplasia and Invasive Squamous Cell Carcinoma of the Cervix Uteri. Clin Cancer Res (2004) 10:3059–63. doi: 10.1158/1078-0432.CCR-03-0565 15131043

[18] Schmitz M Driesch C Beer-Grondke K Jansen L Runnebaum IB Durst M. Loss of Gene Function as a Consequence of Human Papillomavirus DNA Integration. Int J Cancer (2012) 131(5):E593–602. doi: 10.1002/ijc.27433 22262398

[19] Schmitz M Driesch C Jansen L Runnebaum IB Durst M. Non-Random Integration of the HPV Genome in Cervical Cancer. PloS One (2012) 7:e39632. doi: 10.1371/journal.pone.0039632 22761851 PMC3384597

[20] Das P Thomas A Mahantshetty U Shrivastava SK Deodhar K Mulherkar R. HPV Genotyping and Site of Viral Integration in Cervical Cancers in Indian Women. PloS One (2012) 7:e41012. doi: 10.1371/journal.pone.0041012 22815898 PMC3397968

[21] Woodman CB Collins SI Young LS. The Natural History of Cervical HPV Infection: Unresolved Issues. Nat Rev Cancer (2007) 7:11–22. doi: 10.1038/nrc2050 17186016

[22] Luft F Klaes R Nees M Durst M Heilmann V Melsheimer P. Detection of Integrated Papillomavirus Sequences by Ligation-Mediated PCR (DIPS-PCR) and Molecular Characterization in Cervical Cancer Cells. Int J Cancer (2001) 92:9–17. doi: 10.1002/1097-0215(200102)9999:9999<::AID-IJC1144>3.0.CO;2-L 11279600

[23] Klaes R Woerner SM Ridder R Wentzensen N Duerst M Schneider A. Detection of High-Risk Cervical Intraepithelial Neoplasia and Cervical Cancer by Amplification of Transcripts Derived From Integrated Papillomavirus Oncogenes. Cancer Res (1999) 59:6132–6. 10626803

[24] Van Tine BA Kappes JC Banerjee NS Knops J Lai L Steenbergen RD. Clonal Selection for Transcriptionally Active Viral Oncogenes During Progression to Cancer. J Virol (2004) 78:11172–86. doi: 10.1128/JVI.78.20.11172-11186.2004 PMC521852 15452237

[25] Tomlins SA Rhodes DR Perner S Dhanasekaran SM Mehra R Sun XW. Recurrent Fusion of TMPRSS2 and ETS Transcription Factor Genes in Prostate Cancer. Science (2005) 310:644–8. doi: 10.1126/science.1117679 16254181

