# Supplementary material for: Detection of HPV and Human Chromosome Sites by Dual-Color Fluorescence In Situ Hybridization Reveals Recurrent HPV Integration Sites and Heterogeneity in Cervical Cancer
Source: Front Oncol. 2021 Oct 5;11:734758. doi: 10.3389/fonc.2021.734758 (PMC8523950; doi:10.3389/fonc.2021.734758)
Supplement: Supplementary file 1 [file DataSheet_1.docx]

**Protocol for Preparation of FISH Probes by Nick Translation**

1 Template DNA was quantified, and plasmid DNA (1 µg) was used as template DNA.

2 Nick translation system:

| template DNA (plasmid or BAC (Invitrogen)) 1 µg |  |  | X µl |
| --- | --- | --- | --- |
| ultrapure water |  |  | 70-X µl |
| 10×polymerase buffer (Promega) |  |  | 10 µl |
| 1 mM dATP/1 mM dCTP/1 mM dGTP (TaKaRa) |  |  | 3 µl |
| 1 mM dTTP (TaKaRa) |  |  | 2 µl |
| 1 mM biotin dUTP (Promega) or DIG-labeled dUTP (Roche) |  |  | 1 µl |
| 10 U/µl DNA polymerase I (Promega) |  |  | 4 µl |
| 0.002 U/µl DNase I (Promega) |  |  | 10 µl |
|  |  |  | 100 µl |

Polymerase I and DNase I should be added at the end of the system, and the whole process should be performed on ice. The mixture should not be vortexed since vortexing can cause DNase I inactivation. The mixture should be gently mixed with a pipette.

3 Nick translation

The low temperature centrifuge was present to 15°C, and the nick translation mixture was placed in the low-temperature centrifuge for 9~16 hr, usually 13.5 hr in our laboratory.

4 Termination of the nick translation reaction

One microliter of 0.5 mM EDTA solution was added to 100 µl of the nick translation mixture, vortexed and inactivated in a PCR instrument at 80°C for 10 minutes.

5 Determination of the fragment length of the nick translation product by agarose gel electrophoresis

The inactivated nick translation product was placed on ice, and 5 µl was used for 1% agarose gel electrophoresis. The optimal probe size was 200~300 bp and did not exceed 500 bp.

6 The nick translation product can be stored at -80°C for several months.

7 Probe precipitation system:

| nick translation product | 10 µl |
| --- | --- |
| 10 µg/µl ultrasonicated to 500 bp salmon sperm DNA (Invitrogen) | 1 µl |
| 1 µg/µl human cot-1 DNA | 10 µl |
| 3 M pH 5.2 sodium acetate | 2.1 µl (1/10 volume) |
| absolute ethanol | 52.5 µl (2.5 volume) |
|  | 75.6 µl |

The samples were vortexed and frozen at -80°C for more than 2 hours. Then, they were centrifuged at 4°C for 20 minutes. A small amount of DNA precipitate was observed the bottom of the tube. The supernatant was carefully aspirated and discarded. An equal volume of 70% ethanol solution precooled at -80°C was added and centrifuged again at 4°C for 20 minutes, and the supernatant was completely aspirated and discarded. The lid of the tube was opened and air-dried at 50°C for 20 minutes to completely remove the ethanol.

8 Dissolution, denaturation and prehybridization of the probe

Five microliters of deionized formamide solution with pH 7.0 was added and shaken for 20 minutes to dissolve the precipitated DNA. The probe was denatured at 80°C for 10 minutes in the PCR instrument and immediately placed on ice for 1 minute after denaturation was completed. Then, the samples were prehybridized in a PCR instrument at 37°C for 30 min.

9 Next, 5 µl of the master hybridization buffer (mixture of 1 ml of 20 × SSC, 0.5 ml of 20 mg/ml nuclease-free BSA, 1.5 ml of sterile water, 2 ml of 50% (w/v) dextran sulfate) was added to each probe, and the sample was shaken for 20 minutes and stored at -80°C for several months. The probes were then ready for hybridization to target cells.
